# Built-environment attributes associated with refugee children’s physical activity: a narrative review and research agenda

**DOI:** 10.1186/s13031-021-00393-2

**Published:** 2021-07-08

**Authors:** Siqi Chen, Alison Carver, Takemi Sugiyama, Martin Knöll

**Affiliations:** 1 Urban Health Games Research Group (UHGs), Department of Architecture, Technische Universität Darmstadt, Darmstadt, Germany; 2 Mary Mackillop Institute for Health Research, Australian Catholic University, Melbourne, Australia; 3 Centre for Urban Transitions, Swinburne University of Technology, Melbourne, Australia

**Keywords:** Migrants, Outdoor play, Refugee facilities, Micro-environment, Meso-environment, Safety

## Abstract

Research has identified built environmental attributes associated with children’s physical activity (PA); however, less is known for environmental correlates of *refugee* children’s PA. This narrative review summarised the current evidence of associations between built environment attributes and refugee children’s PA. Six databases were searched with three sets of terms related to exposure (built environment); outcome (PA); and target population (refugee children aged 6–12 years). Eight studies (one quantitative; seven qualitative) met the inclusion criteria. Key PA barriers were limited play space and lack of neighbourhood safety. Design of refugee facilities and surrounding environments should provide better access to formal, informal and safe spaces for children’s play.

**Supplementary Information:**

The online version contains supplementary material available at 10.1186/s13031-021-00393-2.

## Introduction

Physical activity (PA) is known to provide health benefits to children [1]. It helps children to build a robust body, stable mental health and healthy relationships with peers [2–4]. Despite the strong evidence supporting the health benefits of PA and public health efforts to promote children’s PA, over 80% children globally do not meet the recommendation of engaging in 60 min of moderate­to­vigorous intensity PA per day [5]. Thus, increasing PA among children is a critical public health goal [6–8].

PA levels appear to be even lower among refugee children, who have recognised refugee status or are asylum seekers [9]. A UNICEF report showed that refugee children were rarely meeting the guidelines for daily PA [10]. Being physically active can be particularly beneficial for refugee children, who have to live in unfamiliar and uncertain situations, which can be stressful [11]. Participation in PA and sport can also help them to build social ties with peers, transcending national boundaries and language barriers [12]. Since refugee children have limited opportunities to engage in organised sports and exercise [13, 14], taking part in informal PA such as active play is particularly important for them [15]. Given that the number of refugees and their children is increasing [16], and that lack of PA can have a long-term impact on children’s health and development [17], it is critical to develop policies and initiatives that can promote PA among refugee children.

There are multiple factors that may be modified to facilitate children to be physically active. One relevant domain is the built environment, which refers to human-made space and structure in which people live, work/study and engages in recreation on a day-to-day basis [18]. Built environmental attributes have been shown to be associated with non-refugee children’s PA. Several literature reviews [19–23] have reported that built environmental attributes such as access to physical activity facilities (playgrounds, greenspaces), availability of sidewalks, neighbourhood perceived safety, and levels of development (urban vs rural) are consistently associated with non-refugee children’s PA.

However, the existing findings of environmental attributes relevant to non-refugee children’s PA may not apply to refugee children. Non-refugee and refugee children live in very different settings. For example, refugee families and their children are typically assigned to refugee camps or other temporary accommodation once they arrive in a host country [24]. Such facilities are often built in isolated and inaccessible areas of cities [25]. Even those who were granted long-term/permanent visa tend to have limited options about where to live and are more likely to reside in disadvantaged areas [26]. Due to such living arrangements, it is possible to argue that refugee children are living in less favourable conditions than non-refugee children for engaging in PA [10]. An increasing number of studies begin to investigate environmental attributes associated with refugee children’s PA. However, to build an evidence base that can inform relevant policies to promote refugee children’s PA, research findings on this topic need to be synthesised.

**Fig. 1:**
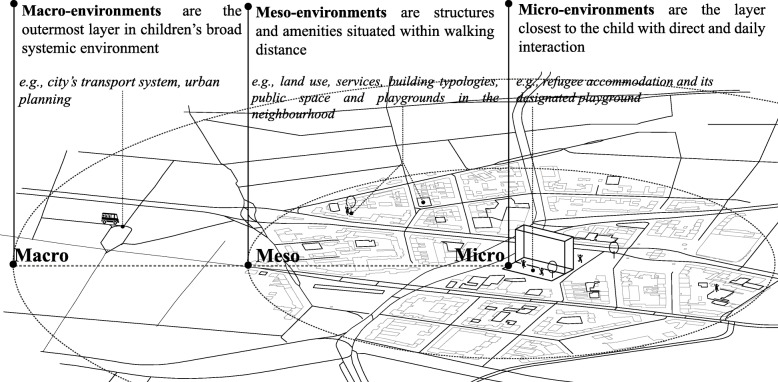
Diagram of environmental attributes on micro-, meso- and macro-level interacting with refugee children’s PA

Bronfenbrenner’s ecological systems theory [27] has been applied as a framework to understand refugee children’s day-to-day activities [28, 29]. The built environment around refugee children includes three environmental layers of interest: *micro-environment; meso-environment; and macro-environment*. The *micro-environment* is the immediate vicinity of the child’s accommodation and contains the structures with which the children have direct contact in their daily lives [29]. Examples include the home/refugee camp and its designated playground [30]. The *meso-environment* is the intermediate layer beyond the immediate surroundings but within the broader neighbourhood including local schools, communities, streets and open spaces. The *macro-environment* involves large-scale features of urban environments such as access to transport infrastructure and regional centres [31]. Figure 1 is a conceptual diagram illustrating these three layers.

The aim of this literature review is to summarise the evidence of associations of micro-, meso-, and macro-built environmental attributes with PA levels among refugee children.

## Methods

### Study search and screening procedures

A systematic search of peer-reviewed publications was conducted by one author (SC) in August 2020. Six electronic databases (PubMed, Web of Science, SPORTDiscus, ERIC, ScienceDirect, and SpringerLink) and one refugee-related journal (Journal of Refugee Studies) were individually searched using three sets of search terms on built environments, physical activity, and the target group. A full description of search queries is shown in Supplementary Material (Table S1). The study selection and screening process was managed using Zotero reference manager software [32]. The articles identified in the search were screened based on their title and abstract first, then based on full text. The initial screening was performed by one author (SC), with randomly selected studies re-evaluated by another author (MK) for consistency. Screening based on full-text articles was carried out by SC, and the results were checked by AC. Any disagreements between them were resolved in consultation with TS. This review was preregistered in PROSPERO (CRD42020201186).

The following inclusion criteria were applied: (1) peer-reviewed journal articles published in English between 2000 and 2020; (2) studies including healthy refugee children and unaccompanied refugee minors aged between 6 and 12 years old; and (3) studies examining associations of built environmental attributes with refugee children’s PA either quantitatively or qualitatively. Articles with a broader age range were considered eligible if they included the 6–12 years age group, and distinct environmental correlates may exist for PA among younger children (2–5 years) [33] and adolescents (13–18 years) [34, 35]. Studies where parents reported children’s PA were also eligible. The review start date of 2000 was chosen, given that refugee children’s physical activity has been examined only recently.

### Data extraction

The following information was extracted from each article: author; publication year; study type (quantitative/qualitative), study design (quantitative only); sample characteristics (size, age, country of origin); study settings (location/host country, length of stay); built environmental attributes (categorised into micro, meso, and macro levels) and measurement methods; PA measures and measurement methods; analysis methods; and findings. Relevant data were extracted, double-checked and all studies were independently appraised by two authors (SC and AC). Any discrepancies were resolved through discussion between them.

### Data synthesis

It was considered that assessing the quality of each study in a formal manner would not add useful information at this stage, due to the fact that research on refugee children’s PA and the built environment is still at an early stage, where most studies are cross-sectional, small scale, and exploratory. For quantitative studies, a relationship between an environmental attribute and a PA measure was considered as a distinct case. A positive relation between them (e.g., more playgrounds related to more PA) was coded “+”, while non-significant relation was coded “0”. Qualitative studies were analysed thematically using NVivo software in three stages: (1) line-by-line coding of primary studies; (2) organising codes into themes and (3) development of analytical themes. Differences in opinion between the reviewers were discussed until consensus was reached. After a full-text evaluation of included studies, a narrative review was chosen due to a small number of eligible articles, most of which were qualitative in design. These reasons also precluded meta-analysis. The final integrated synthesis consists of a narrative commentary for each of three built environment levels and combines the results of quantitative and qualitative syntheses.

## Results

### Characteristics of the studies reviewed

**Fig. 2:**
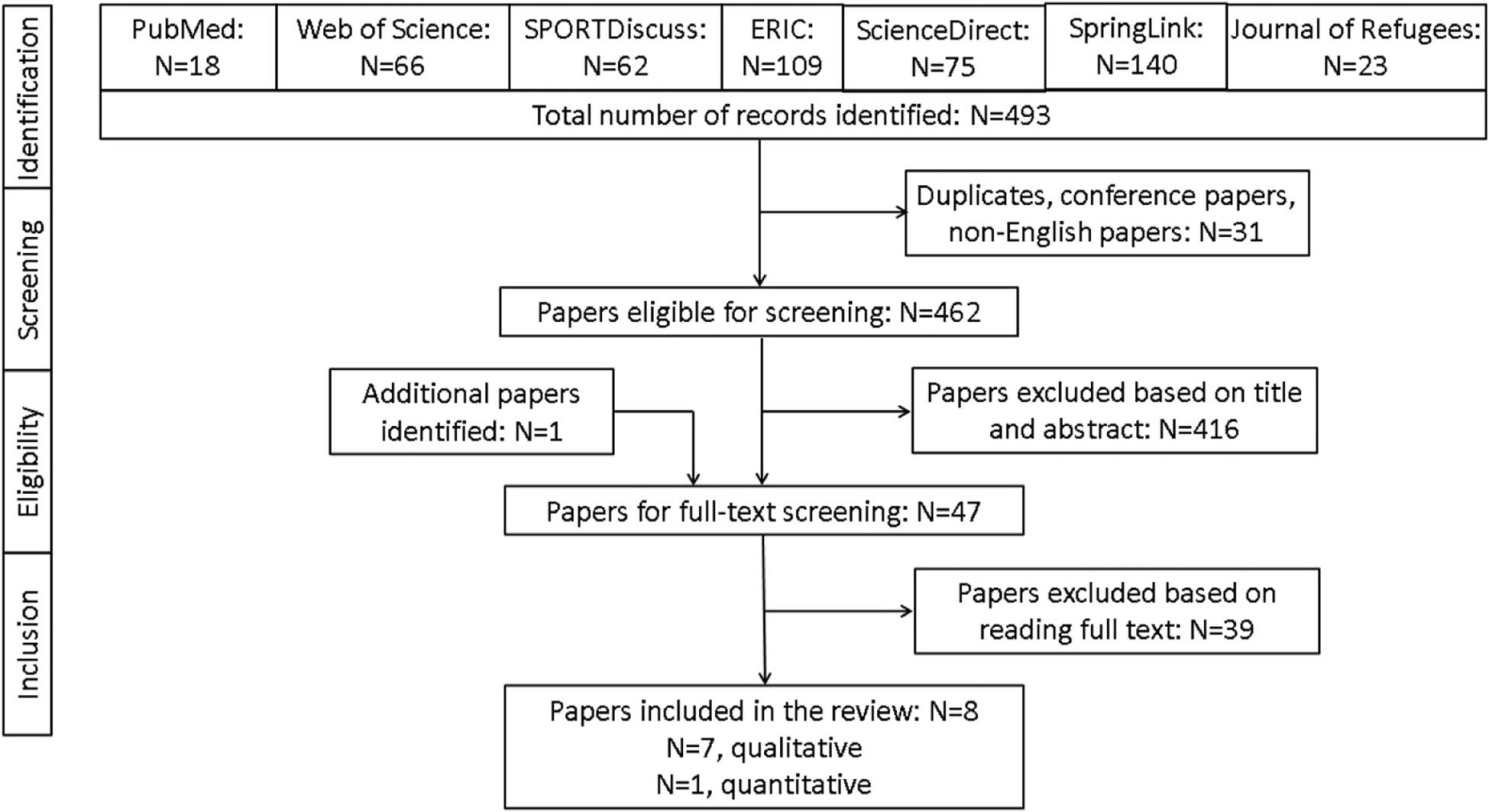
Flow chart of database search and screening

Figure 2 shows the flowchart of the article search/screening process according to the PRISMA (Preferred Reporting Items for Systematic Reviews and Meta-Analyses) statement [36]. A total of 493 studies initially identified were reduced to 47 after screening based on title and abstract. Of these, eight studies (one added at the last stage from authors’ reference lists) remained after the full-text screening. Characteristics of the selected studies are presented in Table 1. Most (75%) of the studies were published in the past five years, and half of them were conducted in the USA. One of the included articles examined a local refugee camp in Palestine [41]. Most of the studies were qualitative, while there was one quantitative study, which observed the number of park users before and after park development for refugees [37]. PA was measured either as self-report or parent-report in 7 studies. One study used observation by researchers [37], while two studies combined observation and self-report measures [39, 41]. Demographics of participants in these studies were as follows: the majority (63%) of the studies investigated children from multi-ethnic backgrounds, and 37% of them came from Muslim countries. Half of the studies examined those with a transit period (in the host country), in which participants spent no more than six months. All of the studies investigated meso-environmental attributes (primarily neighbourhood-level factors), with four studies additionally examining attributes of micro-environments. A detailed description of each study is provided in Supplementary Material (Table S3–4).

**Table 1:** Characteristics of eight studies included in the review

No.	Authors [ref]	Publication year	Study design	Study settings	Countries of origin	Length of stay	Environment-levels	Sample size	PA measurement
1	King et al. [37]	2015	quant.	HIC, USA	Ethnic minority	1–3 years	meso	park observation study	observation
2	Allport et al. [14]	2019	qual.	HIC, UK	*Somali	> 3 years	micro (home), meso	*N* = 6	self- and parent-report
3	Arcan et al. [38]	2018	qual.	HIC, USA	Somali, Latino, Hmong	> 3 years	micro (home), meso	*N* = 67	parent-report
4	Guest [39]	2013	qual.	HIC, USA	No specific, multi-ethnic	< 6 months	meso	*N* = 239 of 380	observation and self-report
5	Hertting & Karlefors [15]	2013	qual.	HIC, Sweden	No specific, multi-ethnic	< 6 months	meso	*N* = 20	self-report
6	MacMillan et al. [40]	2015	qual.	HIC, Australia	*Iran, Indonesia,Pakistan, Malaysia, Kenya, Uganda	< 6 months	meso	*N* = 19	self-report
7	Veronese et al. [41]	2020	qual.	LMIC, Palestine	*Palestine	< 6 months	micro (refugee camp), meso (school, community)	*N* = 29	observation and self-report
8	Wieland et al. [42]	2015	qual.	HIC, USA	Cambodia, Mexico, Somali, Sudan	Not mentioned	micro (home), meso	*N* = 127	self-report

*: Muslim percentage (%) of total population > 70%; qual.: qualitative; quant.: quantitative; HIC: high income countries; LMIC: low- and middle-income countries; “meso” refers to neighbourhood environments unless otherwise specified

### Micro-environment

#### Available indoor space

The *micro-environment*, which refers to refugee children’s home/refugee camp and its immediate vicinity, was examined in four qualitative studies [14, 38, 40, 41]. One factor found to be relevant to PA was the availability of sufficient indoor space for play at home. Two studies [14, 38] reported that cramped living arrangements were a barrier to children playing actively indoors. For example, Somali mothers, who had migrated with their families to Bristol, UK and were residing in small apartments within residential tower blocks, described the lack of individual space and communal facilities within the housing schemes as barriers to their children’s physical activity [14]. Similarly, in a US study [38], Somali, Hmong, and Latino parents who had migrated to Minnesota reported that lack of indoor space in their apartment blocks was a barrier to physical activity. Only one study conducted in a refugee camp setting included a reference to the design of refugee accommodation, and indicated that ‘dedicated spaces’ for play inside the camp (indoors and outdoors) helped children to engage in PA frequently by providing them with a safe environment [41]. There was no quantitative study on micro-environments and refugee children’s PA.

### Meso-environment

The *meso-environment* comprises refugee children’s school/community and broader neighbourhood. All studies reviewed (both quantitative and qualitative) examined meso-environments in relation to refugee children’s PA (Table 2).

**Table 2:** Summary of built-environment attributes associated with refugee children’s PA

Environmental level	Built environmental attributes	Quantitative	Qualitative
		Relationships found	Relationship identified
Micro-environments	Available indoor space		2, 3, 6, 7
	Formal space for PA		7
Meso-environments	Formal space for PA	1 (renovation of play area)	
	Informal space for PA (public, outdoor, green, places for gathering)		2, 3, 5, 6, 7, 8
	Neighbourhood safety (traffic-, sidewalk-organisation, violence)		2, 3, 4, 6, 7
	Accessibility to formal space for PA		2, 3, 4, 6

1: King et al. [37], 2: Allport et al. [14], 3: Arcan et al. [38], 4: Guest [39], 5: Hertting & Karlefors [15], 6: MacMillan et al. [40], 7: Veronese et al. [41], 8: Wieland et al. [42]

#### Formal activity space

It was found that there are two types of activity space relevant to refugee children’s PA. One is ‘formal’, while the other in ‘informal’ activity space (investigated in the next section). In this review, formal space is a play space/area built specifically for the purpose of physical activity, sports and exercise, including playgrounds, basketball courts, and sports fields [14, 37, 38, 42].

A pre- and post-construction observational study [37] investigated refugee children’s physical activity before and after an undeveloped open space adjacent to transitional homes for refugees was transformed into a recreational park. Increased PA was observed in spaces designed for PA after renovation (e.g., play area, ball courts; garden) in children. Moreover, a higher proportion of female children observed within the park post-construction engaged in vigorous physical activity than those observed pre-construction. From the supplementary material provided by the corresponding author, observed cases of girls inside the park boundaries rose from 13 to 79% after the construction. It rose from 35 to 75% for boys. Overall, 85% PA observed in the play area was of moderate to vigorous intensity. Purpose-built play spaces and sports facilities were associated with proportionally more moderate-to-vigorous physical activity and less sedentary behaviour compared with shaded sitting areas. The overall use of adjacent streets, alleys and surrounding parking lots has declined after a park redevelopment.

Limited accessibility to formal space for PA was cited as a negative influence on refugee children’s PA. Qualitative studies reported that limited or lack of access [14, 38] or lack of transportation to exercise facilities [39, 42] were barriers to refugee children’s PA. Moreover, one study indicated that access to outdoor facilities could increase refugee children’s PA [40].

#### Informal activity space

The importance of ‘informal space for PA’ was also a prominent theme that emerged from the qualitative studies. Informal space for PA includes any urban spaces that are readily and freely available by refugee children. Such spaces enable children to engage in physically active, spontaneous play [14, 15, 40, 41]. Children mentioned that there was a lack of space to gather and play as a group, and this appeared to discourage them from engaging in PA [42]. Another study of migrants in the USA reported that refugee children preferred being active in informal gathering spaces with friends rather than engaging in formal sport [42].

#### Safety

Another theme that emerged was neighbourhood safety. Four studies reported that neighbourhoods and school environments need to be safe for refugee children to play [14, 38, 40, 41]. Migrant mothers expressed their concerns about the existing traffic problems and danger from violence in the UK [14]. Since parents considered that adult supervision was required for children’s activities outside, they preferred to keep their children at home [14]. Thus, parents’ safety concern can be a major factor restricting refugee children’s PA.

### Macro-environment

None of the studies included in this review investigated any attributes of *macro-environment,* such as transport systems or urban versus rural areas.

## Discussion

### Summary of research findings

In this review, we identified eight studies examining associations of *micro-* and *meso-environments’* characteristics with refugee children’s PA. Firstly, all but one of the studies were qualitative, and most of them were conducted in the last five years (75%). The empirical research on associations between the built environment and refugee children’s physical activity is in its infancy. Secondly, qualitative studies suggest that both *micro-* and *meso-environments* are relevant to refugee children’s PA. These include available indoor spaces (micro) and accessible formal and informal spaces for PA and safety (meso). One quantitative study found that installing a formal play area in undeveloped greenspace resulted in greater use of that area for PA by refugee children [37]. There was only one study reporting on a low- and middle-income country (LMIC) setting in which children stayed in a temporary refugee camp. All other studies reported on refugee facilities (non-camps) within high-income countries (HICs). Our review shows that studies in LMICs are greatly under-represented, since the majority (68%) of refugees reside in low- and middle-income countries [43].

### Refugee children vs non-refugee children

The authors found that built environmental barriers and facilitators to physical activity for refugee children, i.e., access to physical activity facilities and neighbourhood safety, were similar to those identified for non-refugee children’s PA in earlier reviews. However, the findings do not necessarily mean that refugee and non-refugee children are equal in their access to physical activity facilities. Future research needs to compare refugee and non-refugee children in terms of how active they are, where they engage in physical activity, and how accessible activity spaces are. Such research would highlight the disparities in PA levels and opportunities between refugee and non-refugee children. With regard to safety concerns, they are often about road safety or local crime for non-refugee children [20]. However, refugee children need to adapt to new, unfamiliar environments when they come to their host country. Since they may have escaped from war situations or have experienced military occupation [41], they may be more cautious and sensitive about safety issues than non-refugees [40]. Such concerns by their parents are particularly salient, as where children can play typically dictated by their parents [14]. Future research needs to pay particular attention to how refugee children and parents perceive danger in surrounding environments and to what extent it is different from non-refugee children and parents. This review did not find studies that examined the role of *macro-environment* in refugee children’s PA, although it was found to be related to non-refugee children’s PA [19]. Considering that the location of refugee accommodation is a matter for the discretion of local authorities, future research on this topic is needed to inform where best to build refugee facilities to enhance refugee children’s activity, health and safety.

### Micro- and meso- environments

It was reported that refugee children have limited access to neighbourhood places for their play [14]. In such a situation where meso environments are not conducive to children’s physical activity, *micro-environments* (refugee accommodation and its immediate vicinity) are likely to play an important role in refugee children’s PA in both camps and non-camp settings. However, existing studies on micro-environments do not seem to suggest that refugee facilities provide adequate opportunities for children’s PA. One study reported that being physically active indoors at home is not practical due to noise and space issues [38]. The other study found that passageways, stairwells and basement areas within apartment blocks were utilised as makeshift exercise spaces for occupants [40]. However, they may not be totally safe for children to play. It is recommended that additional spaces suitable for children to be active should be provided in/around their accommodations.

In *meso-environment*s within HICs, one study argued that free access to outdoor space and parks are particularly important for refugee children since their financial situation would not allow them to participate in organised sports and other fee-based activities [14]. However, local parks are not always a safe place to play in deprived areas [44], which are often chosen as a site for refugee accommodation [11]. Given that safety may be a particular concern, research needs to identify what measures can be implemented to ensure parks are safe for refugee children to play. Natural surveillance, in which actions and behaviour in a park can be observed by “eyes on the street”, seems like an important principle [14]. Future studies from HICs can examine other park features (e.g., size, features, distance) that encourage refugee children’s active park use. Only one study was conducted in an LMIC setting [41]. It illustrated that refugee children without access to safe and suitable spaces for PA (e.g., parks) had to use space such as roads, streets and other open spaces despite dangers from military confrontation. Further studies should focus on settings in LMICs to identify PA barriers and facilitators in diverse contexts.

### Formal vs informal spaces for refugee children’s PA

The quantitative study reviewed highlights the importance of formal activity space quality [37]. It found that children’s energy expenditure in park areas increased from 2010 to 2012, after an undeveloped green space park had been transformed into a recreational park with subdivided functional activity zones. It suggests the importance of a high-quality park with suitable facilities and amenities rather than the mere presence of a park. Identifying design attributes of parks relevant to refugee children’s PA is informative for design and management of refugee-related facilities.

Qualitative studies reviewed reported the importance of informal space for refugee children to engage in physical activity [14, 42, 44]. However, this may be a reflection of lack of opportunities for them to take part in sports and exercise. Given that it can be difficult to organise sports in refugee settings, it is important that there is at least informal space such as open spaces where children can be active with friends during leisure time. It is thus conceivable that diverse opportunities (both formal and informal spaces) are important for refugee children’s PA. Considering that participation in sports activities involves not only physical activity but also social interactions, providing refugee children with such opportunities is likely to have multiple benefits [39]. Future studies can assess the effect and feasibility of sports and other activity programs targeting refugee children and investigate their benefits.

### Camp and non-camp settings

The included studies were conducted in different refugee accommodation settings: a refugee camp in an LMIC [41], non-camp settings including designated refugee accommodations located in HICs [14, 15, 37, 39, 40] and community-accommodations specific to their culture in their host countries [38, 42]. It is difficult to compare these settings due to the small number of studies. However, they are likely to differ in terms of the provision of spaces for children. Thus, it could be postulated that environmental correlates of PA may be different for camp and non-camp settings. Further studies should identify environmental attributes related to children’s PA in these diverse contexts, and investigate whether similar environmental attributes may be relevant or there are unique environmental correlates in specific settings.

### Measurement issues for physical activity and built environment

There was no objective measurement of PA in the studies identified. It is evident that self-report measures contain errors and bias in capturing physical activity [45]. Future research needs to employ devices such as accelerometers to measure refugee children’s PA. Furthermore, there was little objective measurement of the built environment in the studies reviewed. The quantitative study by King et al. (2015) provided the pre- and post-construction satellite images, which show the presence of some PA facilities after renovation [37]. The qualitative studies included in this review used self-report measures of the built environment, but these were, by their nature, descriptive and subjective. It is important that further studies employ objectively derived (GIS or audit) measures or validated self-report measures of relevant built environmental attributes. Future studies should learn from existing studies targeting non-refugee children, as they have developed a range of methods to assess the built environment [46]. Particular attention may be given to specific attributes of PA spaces (distance, size, accessibility and features) and safety (perceived safety by parents and by children, objective measures such as crime statistics).

### Gender and cultural differences

Previous studies have shown that refugee girls and boys are likely to play differently [47–49] and have different preferences for places where they would like to play [15, 39, 50]. There was only one study investigating gender differences in this review [37]. It found that more girls participating in vigorous physical activity were observed after park renovation. This seems to suggest that girls may require well-designed places for play, while the presence of open space (without facilities/amenities) may be sufficed for boy’s PA. There were studies examining refugee children from diverse cultural backgrounds [15, 37–40, 42], but they did not examine whether there were between-culture differences in environmental correlates of PA. Further studies need to investigate gender-specific and culture-specific associations between refugee children’s PA and environmental attributes.

### Limitations of the review

There are a few limitations in this review. The inclusion of only peer-reviewed English-language articles may have excluded studies that were conducted in non-English speaking countries with relevant information. For example, much research on refugee children in Germany is reported in German [10, 51]. This review focused on the built environment of places where refugee children lived. However, there may be policies and regulations (e.g., organised PA program) [38, 42] within refugee accommodations, which may be strong determinants of how active children can be. Future reviews may need to consider how policy and environmental factors may be related (independently and jointly) to children’s PA. Finally, we conducted a narrative review, reflecting a small number of studies identified and an early stage of research on this topic. It is expected that more fruitful literature reviews will be conducted in future in light of an increasing interest in refugee’s health and well-being in international contexts.

## Research agenda: recommendations for future studies

This study identified gaps in the literature of environmental attributes associated with PA of school-aged (6–12) refugee children. Overall, this research field requires more quantitative studies to better understand environmental features that are conducive to refugee children’s PA. Below are specific research topics that deserve detailed investigations:

- Examine specific features of environmental attributes (size, quality and accessibility of individual and communal spaces for PA) associated with refugee children’s PA;
- Explore to what extent the quality of formal spaces (presence of physical activity facilities and amenities) and informal spaces (presence of green space and trees, seating, lighting, multiple things to do) are associated with refugee children’s PA;
- Understand the role of macro environments in refugee children’s PA, in particular, whether the location of a refugee accommodation within the city is relevant to their PA levels;
- Use objective measures (i.e., Geographic Information Systems) to identify environmental attributes;
- Identify children’s PA (duration and intensity) using objective measurement methods such as accelerometer;
- Compare environmental correlates of non-refugee and refugee children’s PA in a single study to further understand whether the previous findings on non-refugee children can apply to refugee children;
- Examine environmental correlates of refugee children’s PA in diverse contexts such as in camp and non-camp settings and in low- and middle-income countries;
- Conduct longitudinal studies that track refugee children’s PA patterns when they relocate from a temporary refugee facility to other accommodation;
- Investigate environmental correlates of refugee boys’ and girls’ PA separately to produce gender-specific design recommendations;
- Understand if environmental correlates of refugee children’s PA differ depending on their ethnic backgrounds.

## Conclusion

Children living in refugee accommodation rarely meet physical activity guidelines [10, 51]. This literature review suggests that the built environment where they live (micro and meso environments) is partly contributing to low levels of physical activity. In order to help refugee children to be more physically active, they need to have access to indoor/outdoor play areas in refugee facilities and safe outdoor space for activity in their neighbourhoods. To produce more specific evidence that can inform designs of refugee facilities, more interdisciplinary research involving architecture, urban design, planning, sports science, public health, psychology, and education is necessary. Researchers also need to collaborate with policymakers in refugee-related programs, sports and recreation, and planning to understand their concerns and to disseminate research findings. Given that the number of refugees continues to increase worldwide [52], it is important that host countries provide healthy living environments, particularly for vulnerable groups such as children. Future studies need to build on research of non-refugee children’s physical activity, for which there is a wealth of evidence, to advance our understanding on this topic.

## Supplementary Information

**Additional file 1.**

## Data Availability

The Search strategies and coding is listed in Supplemental material: Table S1; overview of included quantitative studies and qualitative studies are listed in Table S2 and Table S3 and datasets used and/or analysed during the current study are available from the corresponding author on reasonable request.

## Abbreviations

PA: physical activity
UNICEF: United Nations High Commissioner for Refugees
PRISMA: Preferred Reporting Items for Systematic Reviews and Meta-Analyses
HIC: high-income country
LMIC: low- and middle-income country
