# Supplementary material for: Built-environment attributes associated with refugee children’s physical activity: a narrative review and research agenda
Source: Confl Health. 2021 Jul 8;15:55. doi: 10.1186/s13031-021-00393-2 (PMC8268613; doi:10.1186/s13031-021-00393-2)
Supplement: Supplementary file 1 — Additional file 1. [file 13031_2021_393_MOESM1_ESM.doc]

| **Table S1 Search strategies and coding** | | | |
| --- | --- | --- | --- |
| Database | Search coding | Advanced Filters | Search field |
| **PubMed** | ((refugee[Text Word]) OR (asylum seek*[Text Word])) AND ((child*[Text Word]) OR (school age*[Text Word]) OR (minor*[Text Word])) AND ((physical activit*[Text Word]) OR (exercise*[Text Word]) OR (play[Text Word]) OR (sport*[Text Word]) OR (leisure[Text Word]) OR (recreation*[Text Word])) AND ((environment[Text Word]) OR (neighborhood*[Text Word]) OR (neighbourhood*[Text Word]) OR (open space[Text Word]) OR (open spaces[Text Word]) OR (green space[Text Word]) OR (green spaces[Text Word]) OR (park[Text Word]) OR (parks[Text Word]) OR (playland[Text Word]) OR (playlands[Text Word]) OR (playground[Text Word]) OR (playgrounds[Text Word]) OR (sport field[Text Word]) OR (play fields[Text Word]) OR (sport ground[Text Word]) OR (sport grounds[Text Word]) OR (facility[Text Word]) OR (facilities[Text Word]) OR (gym[Text Word]) OR (gyms[Text Word])) | 1996-2020, Humans, English | full text |
| **Web of Science** | ((AB = "physical* activit*") OR (AB="vigorous* activit*") OR (AB= exercise) OR (AB="active transport*") OR (AB=play) OR (AB=walking) OR (AB=sport*) OR (AB=fitness) OR (AB="energy expenditure") OR (AB=leisure) OR (AB=outdoor) OR (AB=recreation*)) AND ((AB=refugee*) OR (AB=asylum seek*)) AND ((AB=child*) OR (AB=minor*) OR (AB=school age*)) AND ((AB=environment*) OR (AB=neighbo$rhood) OR (AB=open space*) OR (AB="green space") OR (AB="green spaces") OR (AB=park*) OR (AB=parkland) OR (AB=playground*) OR (AB=playtime) OR (AB=sport* field) OR (AB=sport* ground) OR (AB=facilit*) OR (AB=gym*)) | 1996-2020, English, peer-reviewed | abstract |
| **SPORT Discus** | TX/AB ( refugee OR asylum seek* ) AND TX ( child* OR minor OR school age* ) AND TX ( physical activit* OR exercise OR play OR sport OR leisure OR recreation* ) AND TX ( environment OR neighborhood OR neighbourhood OR open space OR park OR playland OR playground OR sport field OR play field OR sport ground OR facilit OR gym) | 1996-2020, English, peer-reviewed Search modes - Boolean/Phrase | abstract and full text |
| **ERIC** | (ab(refugee*) OR ti(refugee*) OR ab(asylum seek*) OR ti(asylum seek*)) AND (ab(child*) OR ti(child*) OR ab(minor*) OR ti(minor*) OR ab(school age*) OR ti(school age*)) AND (ab(physical activit*) OR ti(physical activit*) OR ab(exercise) OR ti(exercise) OR ab(play) OR ti(play) OR ab(sport*) OR ti(sport*) OR ab(leisure) OR ti(leisure) OR ab(recreation*) OR ti(recreation*)) AND (ab(environment*) OR ti(environment*) OR ab(neighborhood*) OR ti(neighborhood*) OR ab(neighbourhood*) OR ti(neighbourhood*) OR ab(open space*) OR ti(open space*) OR ab(park*) OR ti(park*) OR ab(playland*) OR ti(playland*) OR ab(playground*) OR ti(playground*) OR ab(sport* field*) OR ti(sport* field*) OR ab(play field*) OR ti(play field*) OR ab(sport ground*) OR ti(sport ground*) OR ab(facilit*) OR ti(facilit*) OR ab(gym*) OR ti(gym*)) AND (pubyear:1996-2020) AND (LA(English)) | Peer-reviewed | title and abstract |
| **ScienceDirect** | FT(refugee OR asylum seek) AND (child OR minor OR school age) AND TX (physical activit OR exercise OR play OR sport OR leisure OR recreation) AND (environment OR neighborhood OR neighbourhood OR open space/ OR park OR playland OR playground/ OR sport field OR play field OR sport ground/ OR facilit OR gym) | 1996-2020, peer reviewed | title and abstract and full text |
| **SpringerLink** | (refugee OR asylum seek*) AND (child* OR minor OR school age*) AND (physical activit OR vigorous activit OR exercise OR active transport OR play OR walking OR sport OR fitness OR energy expenditure OR leisure OR outdoor OR recreation) AND (environment OR neighborhood OR neighbourhood OR open space OR park OR playland OR playground OR playtime OR sport field OR play field OR sport ground OR facilit OR gym) | 1996-2020, English | full-text |
| **Journal of Refugee studies** | FT (child* OR minor OR school age*) AND (physical activit OR exercise OR play OR sport OR leisure OR recreation) AND (environment OR neighborhood OR neighbourhood OR open space OR park OR playland OR playground OR sport field OR play field OR sport ground OR facilit OR gym) | 1996-2020 | full text and any field |

| **Table S2 Overview of quantitative study** | | | | | | | | |
| --- | --- | --- | --- | --- | --- | --- | --- | --- |
| **No.** | **Authors and locations** | **Environment-levels** | **Countries of origin** | **Sample** | **PA Intervention/ Exposure variable** | **Study design and PA measurement** | **Data analysis methods and PA levels** | **Findings associated with PA** |
| 1 | **King et al., 2015, USA** | meso (neighbourhood) | Ethnic minority | (2010, N= 1530, 2012, N=1946), under 12 years | Undeveloped green space park transformed into a recreational park | Prospective non-randomised design using System of Observing Play and Recreation in Communities (SOPARC). Activity levels were categorised as sedentary (lying down, sitting or standing), moderate (casual walking) and vigorous (expending more energy than casual walking). PA codes were converted to energy expenditure (kcal/kg/min). Total energy expenditure (EE) scores in different park areas were calculated by multiplying totals observed in sedentary, moderate, or vigorous activity by 0.051 kcal/kg/min; 0.096 kcal/kg/min; or 0.144 kcal/kg/min, respectively. | T-tests or tests of medians (when appropriate) were used to compare pre- and post-construction changes in use of non-park and park zones for PA by age-group and gender. | • More female children engaged in vigorous activity • A increase in total energy expended inside the park boundaries among boys and girls • A decline in total energy expended on adjacent streets, alleys and surrounding parking lots |
| **Table S3 Overview of qualitative studies** | | | | | | | | |
| **No.** | **Authors and locations** | **Environment-levels** | **Countries of origin** | **Sample** | **Objectives of study** | **Study design/PA report methods** | **Data analysis methods and PA types** | **Findings - factors influencing PA** |
| 2 | **Allport et al., 2019, UK** | micro (home), meso (neighbourhood) | Somali | (N= 6), mothers (the mothers were 6-8 years when they left their countries of origin) | To explore the geography of childhood from the perspective of Somali mothers who have resettled in Bristol. | Semi-structured interviews | Interpretative phenomenological approach, **play related to space** | A decline in **accessible outdoor public space** and **fears about traffic** in UK compared with Somalia may reduce opportunities for free play. Mothers felt that their children's play was constrained by **living in tower blocks (social housing) with few communal facilities**. |
| 3 | **Arcan et al., 2018, USA** | micro (home), meso (neighbourhood) | Somali, Latino, Hmong | (N= 67) parents of children aged 3-12 years | To identify perceptions of childhood bodyweight and approaches to raising healthy children | 10 focus groups | Thematic analysis with CBPR principles, **PA** | Parents thought interventions (**e.g., safe places to be active**) could help them with children's Physical inactivity |
| 4 | **Guest, 2013, USA** | meso (neighbourhood) | No specific, multi-ethnic | (N= 380, Concrete Park:141, M 8.96 years, SD 1.86, Pena: 239, M 9.61 years, SD 1.75s), 6-12 years. Only the children in Pena were refugees. | To investigate the meanings of informal sport and play to childhood | Direct observations, interviews and ethnographic anecdotes | ethnographic methodology, **informal sports and play** | The important of **informal space** for refugee children's PA |
| 5 | **Hertting & Karlefors, 2013, Sweden** | meso (neighbourhood) | No specific, multi-ethnic | (N= 20) 10-13 years | To explore images and experiences that refugee children have about sport in their country of origin, and challenges that can arise in processes of integration through sport | Drawings and oral comments | phenomenology, **sport experience** | • Taking advantage of **informal space** could promote refugee children's PA.  • **Formal sports facilities** in their former home countries were associated with more serious organised sports and more pressure to perform well. |
| 6 | **MacMillan et al., 2015, Australia** | meso (neighbourhood) | Iran, Indonesia, Pakistan, Malaysia,  Kenya, Uganda | (N= 19, M 8.5 years SD 6.4 months) 8-10 years old | To explore how refugee children engaged in play pre-migration and post-migration to Australia. | Drawings and interviews | Drawings were coded and analysed using cross-tabulation to compare pre- and post-migration **play** | • Through their drawings, significantly fewer children reported playing pre- versus post-migration (58% vs 95%, P < 0.03).  • Girls had more significant relative changes in play with migration (pre: 25% vs post: 87%). **Almost all play was outdoors** (pre: 91%; post: 94.4%).  • Perceived **lack of safety** was reported as a barrier to pre-migration play. |
| 7 | **Veronese et al., 2020, Palestine** | micro (refugee camp), meso (school, neighbourhood) | Palestine | (N= 29) 7-13 years (3 children of 7 years old, 3 of 8 years old, 7 of 9 years old, 9 of 10 years old, 3 of 11 years old, 2 of 12 years old, 2 of 13 years old). | To explore the sources of spatial agency that children draw on to counteract the harmful consequences of ongoing exposure to trauma | Drawings and walk-along interviews | place-based method, **play** | • internal spaces as a **safe place f**or growing and developing **•community spaces as places** where children have fun and play an active role, and inhabiting the outdoor spaces in the camp despite environmental dangers and the occupation. |
| 8 | **Wieland et al., 2015, USA** | micro (home), meso (neighbourhood) | Cambodia, Mexico, Somali, Sudan | (N= 127) adults and children 11-18 years old (unclear how many of each) | To explore the reason that immigrants and refugees to the United States exhibit relatively low levels of physical activity | 16 gender and age-stratified focus groups | Thematic analysis, **PA** | **Lack of familiarity with and comfort in the environment** that hinder the taking the first steps towards being physically active were the most significant barriers to PA. There is little reference to the built environment except for lack of transport to exercise facilities and lack of spaces for groups to gather for affordable PA opportunities. |
